# Comprehensive Analysis of Adverse Events Associated With SGLT2is: A Meta-Analysis Involving Nine Large Randomized Trials

**DOI:** 10.3389/fendo.2021.743807

**Published:** 2021-12-02

**Authors:** Mei Qiu, Li-Min Zhao, Ze-Lin Zhan

**Affiliations:** ^1^ Department of General Medicine, Shenzhen Longhua District Central Hospital, Shenzhen, China; ^2^ Class 3, Clinical Medicine, Grade 2019, The Second Clinical Medical College, Southern Medical University, Guangzhou, China

**Keywords:** SGLT2is, safety, cardiovascular diseases, renal diseases, asthma, pneumonia, sleep apnoea syndrome, chronic obstructive pulmonary disease

## Abstract

Recently, Lin and colleagues assessed the safety of sodium-glucose cotransporter 2 inhibitors (SGLT2is) by a meta-analysis [1], in which the authors assessed 16 kinds of adverse events (AE) reported in the published articles based on 10 randomized controlled trials. We conducted a further meta-analysis and targeted the association between use of SGLT2is and occurrences of various kinds of serious AE published in the Clinical Trials website (clinicaltrials.gov). Our meta-analysis revealed that use of SGLT2is was not significantly associated with occurrences of 980 kinds of serious AE but was significantly associated with lower risks of 29 kinds of serious AE, especially including several important respiratory diseases (e.g., asthma, chronic obstructive pulmonary disease, sleep apnoea syndrome, and pneumonia). These findings may cause more studies to evaluate the possibilities of gliflozins being used for prevention of these specific diseases.

## Introduction

In a meta-analysis (1) recently published in *Journal of Clinical Endocrinology & Metabolism*, Lin and colleagues investigated the safety of sodium-glucose cotransporter 2 inhibitors (SGLT2is) by assessing 16 kinds of clinical adverse events (AE) associated with SGLT2is. Accordingly, the authors drew their main conclusion that use of SGLT2is was generally safe. Although those AE assessed in the meta-analysis by Lin et al. (1) included four overall safety outcomes (e.g., AE, and serious AE) and 12 specified safety outcomes (e.g., genital infections, and diabetic ketoacidosis), all of them are only limited to the AE that were reported in the published articles based on 10 randomized controlled trials (RCTs) (2–11) included in the meta-analysis by Lin et al. (1). However, Lin et al. did not assess hundreds of kinds of AE which were published in the Clinical Trials website (clinicaltrials.gov) a few months after the articles reporting RCTs were published in relevant journals. Hence, we conducted a further meta-analysis and targeted the association between SGLT2is and those various AE that were published in the Clinical Trials website, in order to verify the main conclusion of Lin et al. (1) and possibly propose novel viewpoints regarding the effects of SGLT2is on safety outcomes.

## Methods

The 10 large RCTs (2–11) included in the meta-analysis by Lin et al. (1) were CREDENCE trial (2), CANVAS Program trial (3), DECLARE-TIMI 58 trial (4), EMPA-REG OUTCOME trial (5), VERTIS CV trial (6), EMPEROR-Reduced trial (7), DAPA-HF trial (8), DAPA-CKD trial (9), SOLOIST-WHF trial (10), and SCORED trial (11). The former eight trials (2–9) had reported in detail the data of various AE in the Clinical Trials website (clinicaltrials.gov) before the data extraction for this meta-analysis began (the date was June 25, 2021), whereas the latter two trials (10, 11) had not reported. Therefore, in this meta-analysis we could only incorporate the relevant safety data from the former eight trials (2–9). Since the CANVAS Program trial (3) consisted of the CANVAS trial (NCT01032629) (3) and the CANVAS-R trial (NCT01989754) (3), this meta-analysis actually incorporated the AE data which were reported in the Clinical Trials website by nine trials, namely, CANVAS trial (3), CANVAS-R trial (3), and seven other trials (2, 4–9).

Although we extracted all the data regarding serious AE which were reported by included trials in the Clinical Trials website at first, not all of the serious AE were analyzed in this meta-analysis. On the contrary, meta-analysis was done on a certain safety outcome only when this safety outcome was reported by at least three of the included trials. The reasons for doing this are as follows. First, according to statistical requirement, meta-analysis can be conducted only when at least two studies are included. Second, occurrences of various serious AE reported in the Clinical Trials website were low, and therefore to guarantee essential statistical power we performed meta-analysis only when at least three trials were involved. According to this criterion, among all the serious AE reported in the Clinical Trials website 1,009 kinds of serious AE were identified for further meta-analyses. These 1,009 safety outcomes involved various disorders in 24 body systems (listed in **Tables SPI:** **S1**–**S24**), including 1) blood and lymphatic system disorders, 2) cardiac disorders, 3) congenital, familial, and genetic disorders, 4) ear and labyrinth disorders, 5) endocrine disorders, 6) eye disorders, 7) gastrointestinal disorders, 8) general disorders, 9) hepatobiliary disorders, 10) immune system disorders, 11) infections and infestations, 12) injury, poisoning, and procedural complications, 13) investigations, 14) musculoskeletal and connective tissue disorders, 15) neoplasms benign, malignant, and unspecified, 16) nervous system disorders, 17) product issues, 18) psychiatric disorders, 19) renal and urinary disorders, 20) reproductive system and breast disorders, 21) respiratory, thoracic, and mediastinal disorders, 22) skin and subcutaneous tissue disorders, 23) surgical and medical procedures, and 24) vascular disorders. We did not assess those disorders regarding the metabolism and nutrition system because they had already been well investigated.

We conducted meta-analyses using the fixed-effect inverse variance method to estimate pooled risk ratios (RRs) and 95% confidence intervals (CIs). We measured the magnitude of heterogeneity *via* the I^2^ statistic. Statistical analyses were implemented in the Stata/MP software (version 16.0).

## Findings

**Figure 1:**
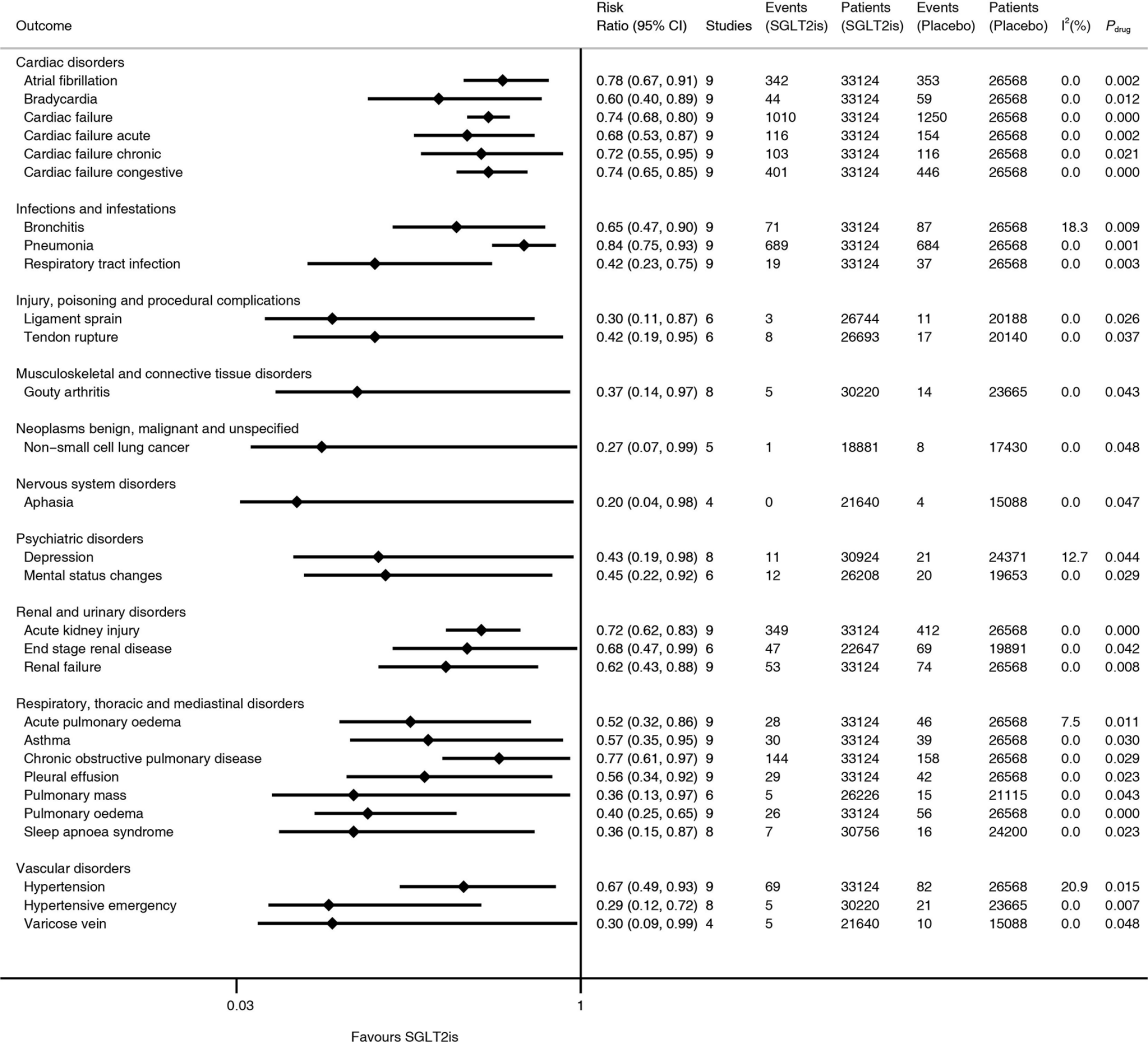
Meta-analyses of SGLT2is and 29 kinds of serious adverse events. SGLT2is, sodium-glucose cotransporter 2 inhibitors; CI, confidence interval; *p*_drug_, p for drug effect.

Nine trials (2–9) included in this meta-analysis involved a total of 33,124 subjects in the SGLT2i group and 26,568 subjects in the placebo group, and all the included studies were with low risk of bias. As is shown in **Figure 1**, compared with placebo SGLT2is were significantly associated with lower risks of 29 kinds of serious AE (i.e., atrial fibrillation [RR 0.78], bradycardia [RR 0.60], cardiac failure [RR 0.74], cardiac failure acute [RR 0.68], cardiac failure chronic [RR 0.72], cardiac failure congestive [RR 0.74], bronchitis [RR 0.65], pneumonia [RR 0.84], respiratory tract infection [RR 0.42], ligament sprain [RR 0.30], tendon rupture [RR 0.42], gouty arthritis [RR 0.37], non-small cell lung cancer [RR 0.27], aphasia [RR 0.20], depression [RR 0.43], mental status changes [RR 0.45], acute kidney injury [RR 0.72], end-stage renal disease [RR 0.68], renal failure [RR 0.62], acute pulmonary oedema [RR 0.52], asthma [RR 0.57], chronic obstructive pulmonary disease [RR 0.77], pleural effusion [RR 0.56], pulmonary mass [RR 0.36], pulmonary oedema [RR 0.40], sleep apnoea syndrome [RR 0.36], hypertension [RR 0.67], hypertensive emergency [RR 0.29], and varicose vein [RR 0.30]). The lower limits of 95% CIs of RRs ranged from 0.04 to 0.75, while the upper limits ranged from 0.65 to 0.99. p values for drug effect ranged from <0.001 to 0.048, and most of the I^2^ values were equal to 0. The above 29 kinds of serious AE involved 10 body systems of disorders (i.e., 1) cardiac disorders, 2) infections and infestations, 3) injury, poisoning, and procedural complications, 4) musculoskeletal and connective tissue disorders, 5) neoplasms benign, malignant, and unspecified, 6) nervous system disorders, 7) psychiatric disorders, 8) renal and urinary disorders, 9) respiratory, thoracic, and mediastinal disorders, and 10) vascular disorders).

In contrast, SGLT2is versus placebo were not significantly associated with occurrences of the other 716 kinds of serious AE in the above 10 body systems and were also not significantly associated with occurrences of 264 kinds of serious AE in the other 14 body systems (these 14 body systems of disorders consisted of 1) blood and lymphatic system disorders, 2) congenital, familial, and genetic disorders, 3) ear and labyrinth disorders, 4) endocrine disorders, 5) eye disorders, 6) gastrointestinal disorders, 7) general disorders, 8) hepatobiliary disorders, 9) immune system disorders, 10) investigations, 11) product issues, 12) reproductive system and breast disorders, 13) skin and subcutaneous tissue disorders, and 14) surgical and medical procedures). The summary results of meta-analyses on 1,009 kinds of serious AE in 24 body systems are presented in **Tables S1**–**S24**.

## Discussion

According to several previous meta-analyses (1, 12–18) focusing on the safety of SGLT2is, there are relatively consistent findings regarding the association of SGLT2is with several kinds of AE as follows. SGLT2is increased the risks of diabetic ketoacidosis (1, 12, 13), genital infections (1, 12, 14), and volume depletion (1, 12) and probably increased urinary tract infection risk (1, 12), whereas they reduced acute kidney injury risk (1, 12). Moreover, a specific SGLT2i canagliflozin was associated with an increased risk of amputation (15, 16). Oppositely, the fracture risk of SGLT2is is a controversial issue. A meta-analysis (17) found that SGLT2is had no effect on fractures, whereas one other meta-analysis (12) revealed that SGLT2is had an increased trend in fracture risk. Meanwhile, Zhang and colleagues (18) thought that some SGLT2is such as empagliflozin and ertugliflozin might increase the risk of fracture whereas others such as canagliflozin and dapagliflozin might reduce that risk. Therefore, there is a need for further investigation regarding this issue.

Different with these above common AE associated with SGLT2is, those AE this present meta-analysis focused on were the various serious AE reported in the Clinical Trials website, which were rare, and were various specific AE rather than summary AE. For example, we assessed the risks of dozens of kinds of site-specific fractures (as shown in **Table S12**), whereas previous meta-analyses (12, 17, 18) assessed only the risk of overall fractures. This is one of the innovative points in our meta-analysis. Moreover, compared with several previous meta-analyses (1, 12–18) assessing the safety of SGLT2is by analyzing a limited number of safety outcomes, our meta-analysis is the first one that analyzed hundreds of safety outcomes associated with SGLT2is. Accordingly, it revealed that use of SGLT2is was not significantly associated with occurrences of 980 kinds of serious AE but was significantly associated with lower risks of 29 kinds of serious AE. Apart from confirming the main conclusion of Lin et al. that use of SGLT2is was generally safe (1), our findings have several other implications. First of all, SGLT2is were observed with lower risks of several important respiratory diseases including asthma, chronic obstructive pulmonary disease, sleep apnoea syndrome, and pneumonia in this meta-analysis. This will cause further studies to assess whether this drug class could be used to prevent these respiratory diseases.

Second, in terms of cardiac and renal systems, our meta-analysis showed a significant association of SGLT2is with lower risks of cardiac failure and renal failure. This is consistent with the cardiorenal benefits of SGLT2is revealed by the analyses on primary outcomes in cardiorenal outcome trials. Moreover, our meta-analysis also revealed a significant association of SGLT2is with lower risks of bradycardia and atrial fibrillation. This finding may suggest the possible antiarrhythmic efficacy of SGLT2is.

Third, this meta-analysis revealed significant association of SGLT2is with lower risks of three kinds of vascular diseases (i.e., hypertension, hypertensive emergency, and varicose vein) and three kinds of infectious diseases (i.e., pneumonia, bronchitis, and respiratory tract infection). SGLT2is were observed with lower risks of hypertension and hypertensive emergency, which is probably because SGLT2is improve the bioavailability of endothelium-derived nitric oxide, favourably regulate the activity of endothelial cells, suppress the contraction of vascular smooth muscle cells (19), and reduce the activity of sympathetic nervous system (20). Meanwhile, their glucose-lowering efficacy may be associated with their lower risks of respiratory infections.

Last, some statistically significant findings should be interpreted with caution due to those wide 95% CIs of RR, such as the significant association between use of SGLT2is and occurrences of gouty arthritis, non-small cell lung cancer, and depression. These need to be confirmed by future studies with a greater number of events.

The main weakness of this study is that all the AE assessed in this meta-analysis were the serious AE published in the Clinical Trials website (clinicaltrials.gov), rather than the prespecified AEs reported in the published articles. Moreover, most of the AE assessed in this meta-analysis were infrequent, which led to the limited statistical power in corresponding data analyses. Therefore, the significant association relationships between SGLT2is and lower risks of multiple serious AE revealed in this meta-analysis could be only considered as hypothesis-generating findings, but not conclusive causal relationships. Further validation is essential.

In conclusion, this meta-analysis revealed that use of SGLT2is was not associated with increased risks of 1,009 kinds of serious AE in 24 body systems, which confirms the main conclusion of Lin et al. that use of SGLT2is was generally safe (1). Moreover, it revealed that use of SGLT2is was associated with lower risks of 29 kinds of serious AE, especially including several important respiratory diseases (e.g., asthma, chronic obstructive pulmonary disease, sleep apnoea syndrome, and pneumonia). These findings may cause more studies to evaluate the possibilities of gliflozins being used for prevention of these specific diseases.

## Author Contributions

Design: MQ. Data curation: MQ, L-MZ, and Z-LZ. Analysis: L-MZ and MQ. Writing—original draft: MQ. Writing—review and editing: L-MZ and Z-LZ. All authors contributed to the article and approved the submitted version.

## Conflict of Interest

The authors declare that the research was conducted in the absence of any commercial or financial relationships that could be construed as a potential conflict of interest.

## Publisher’s Note

All claims expressed in this article are solely those of the authors and do not necessarily represent those of their affiliated organizations, or those of the publisher, the editors and the reviewers. Any product that may be evaluated in this article, or claim that may be made by its manufacturer, is not guaranteed or endorsed by the publisher.

## Supplementary Material

Supplementary Table 1 Meta-analyses of SGLT2is and 1009 kinds of serious adverse events (the results are shown according to 24 different body systems.

Supplementary Table 2 Meta-analyses of SGLT2is and 1009 kinds of serious adverse events (the results are shown according to 24 different body systems.

Supplementary Table 3 Meta-analyses of SGLT2is and 1009 kinds of serious adverse events (the results are shown according to 24 different body systems.

Supplementary Table 4 Meta-analyses of SGLT2is and 1009 kinds of serious adverse events (the results are shown according to 24 different body systems.

Supplementary Table 5 Meta-analyses of SGLT2is and 1009 kinds of serious adverse events (the results are shown according to 24 different body systems.

Supplementary Table 6 Meta-analyses of SGLT2is and 1009 kinds of serious adverse events (the results are shown according to 24 different body systems.

Supplementary Table 7 Meta-analyses of SGLT2is and 1009 kinds of serious adverse events (the results are shown according to 24 different body systems.

Supplementary Table 8 Meta-analyses of SGLT2is and 1009 kinds of serious adverse events (the results are shown according to 24 different body systems.

Supplementary Table 9 Meta-analyses of SGLT2is and 1009 kinds of serious adverse events (the results are shown according to 24 different body systems.

Supplementary Table 10 Meta-analyses of SGLT2is and 1009 kinds of serious adverse events (the results are shown according to 24 different body systems.

Supplementary Table 11 Meta-analyses of SGLT2is and 1009 kinds of serious adverse events (the results are shown according to 24 different body systems.

Supplementary Table 12 Meta-analyses of SGLT2is and 1009 kinds of serious adverse events (the results are shown according to 24 different body systems.

Supplementary Table 13 Meta-analyses of SGLT2is and 1009 kinds of serious adverse events (the results are shown according to 24 different body systems.

Supplementary Table 14 Meta-analyses of SGLT2is and 1009 kinds of serious adverse events (the results are shown according to 24 different body systems.

Supplementary Table 15 Meta-analyses of SGLT2is and 1009 kinds of serious adverse events (the results are shown according to 24 different body systems.

Supplementary Table 16 Meta-analyses of SGLT2is and 1009 kinds of serious adverse events (the results are shown according to 24 different body systems.

Supplementary Table 17 Meta-analyses of SGLT2is and 1009 kinds of serious adverse events (the results are shown according to 24 different body systems.

Supplementary Table 18 Meta-analyses of SGLT2is and 1009 kinds of serious adverse events (the results are shown according to 24 different body systems.

Supplementary Table 19 Meta-analyses of SGLT2is and 1009 kinds of serious adverse events (the results are shown according to 24 different body systems.

Supplementary Table 20 Meta-analyses of SGLT2is and 1009 kinds of serious adverse events (the results are shown according to 24 different body systems.

Supplementary Table 21 Meta-analyses of SGLT2is and 1009 kinds of serious adverse events (the results are shown according to 24 different body systems.

Supplementary Table 22 Meta-analyses of SGLT2is and 1009 kinds of serious adverse events (the results are shown according to 24 different body systems.

Supplementary Table 23 Meta-analyses of SGLT2is and 1009 kinds of serious adverse events (the results are shown according to 24 different body systems.

Supplementary Table 24 Meta-analyses of SGLT2is and 1009 kinds of serious adverse events (the results are shown according to 24 different body systems.

## Abbreviations

SGLT2is, sodium-glucose cotransporter 2 inhibitors; AE, adverse events; RR, risk ratio; CI, confidence interval
